# The m^6^A Reader YTHDF1 Facilitates the Tumorigenesis and Metastasis of Gastric Cancer via USP14 Translation in an m^6^A-Dependent Manner

**DOI:** 10.3389/fcell.2021.647702

**Published:** 2021-03-15

**Authors:** Xiao-Yu Chen, Rui Liang, You-Cai Yi, Hui-Ning Fan, Ming Chen, Jing Zhang, Jin-Shui Zhu

**Affiliations:** Department of Gastroenterology, Shanghai Jiao Tong University Affiliated Sixth People’s Hospital, Shanghai, China

**Keywords:** tumorigenesis, N^6^-methyladenosine, metastasis, gastric cancer, YTHDF1, USP14

## Abstract

**Objectives:**

N^6^-methyladenosine (m^6^A) RNA methylation is implicated in the progression of multiple cancers via influencing mRNA modification. YTHDF1 can act as an oncogene in gastric cancer (GC), while the biological mechanisms via which YTHDF1 regulates gastric tumorigenesis through m^6^A modification remain largely unknown.

**Methods:**

GEO and TCGA cohorts were analyzed for differentially expressed m^6^A modification components in GC clinical specimens and their association with clinical prognosis. Transwell and flow cytometry assays as well as subcutaneous xenograft and lung metastasis models were used to evaluate the phenotype of YTHDF1 in GC. Intersection of RNA/MeRIP-seq, luciferase assay, RIP-PCR, RNA pull-down and MeRIP-PCR was used to identify YTHDF1- modified USP14 and its m^6^A levels in GC cells.

**Results:**

High-expressed YTHDF1 was found in GC tissues and was related to poor prognosis, acting as an independent prognostic factor of poor survival in GC patients. YTHDF1 deficiency inhibited cell proliferation and invasion (*in vitro*), and gastric tumorigenesis and lung metastasis (*in vivo*) and also induced cell apoptosis. Intersection assays revealed that YTHDF1 promoted USP14 protein translation in an m^6^A-dependent manner. USP14 upregulation was positively correlated with YTHDF1 expression and indicated a poor prognosis in GC.

**Conclusion:**

Our data suggested that m^6^A reader YTHDF1 facilitated tumorigenesis and metastasis of GC by promoting USP14 protein translation in an m^6^A-dependent manner and might provide a potential target for GC treatment.

## Introduction

According to the global cancer statistics, gastric cancer (GC) is regarded as one of the most invasive cancers and is the third leading factor of tumor-associated deaths (Bray et al., 2018). Past decades have witnessed great progress in diagnostic and therapeutic methods of GC including targeted therapy, and immunotherapy (Ajani et al., 2017). However, the prognosis of patients with GC, especially metastatic cases, is still unfavorable due to the aggressiveness of the tumor. Hence, we urgently need to obtain novel biomarkers for early screening and treatment of GC.

N^6^-methyladenosine (m^6^A) is a member of chemical modifications commonly and abundantly found in eukaryotic mRNAs, and its modification is a dynamic and reversible process (Jia et al., 2011). The m^6^A RNA modification is initiated by the m^6^A methyltransferases complex (“writers”) including METTL3/14, WTAP, RBM15/15B, and KIAA1429, reversed by demethylases (FTO and ALKBH5, named “erasers”), and identified by m^6^A binding proteins (YTHDF1/2/3, IGF2BP1, HNRNPA2B1, and BCDIN3D, termed as “readers”) (Chen et al., 2019). Recent studies have indicated that the m^6^A modification is involved in tumor progression and acts as tumor-promoting or suppressive factors in multiple malignancies (Lin et al., 2016; Ma et al., 2017; Liu J. et al., 2018; Chen et al., 2019). METTL3 enhances metastasis of GC by m^6^A modification of ZMYM1/E-cadherin signaling, and WTAP results in a poor clinical prognosis of GC through regulating tumor-related immune infiltration (Yue et al., 2019; Li H. et al., 2020). YTHDF1 (m^6^A “reader”) can be used to predict poor prognosis in breast cancer and promotes FDZ5 translation, leading to hepatocellular carcinoma progression (Anita et al., 2020; Liu X. et al., 2020). YTHDF1 can bind to mammalian mRNAs and alter the protein translation process in a m^6^A-dependent manner. For example, it can modulate the translational efficiency of cyclin-dependent kinases in lung adenocarcinomas (Shi et al., 2019), EIF3C in ovarian cancer (Liu T. et al., 2020), and lysosomal cathepsins in melanoma and colorectal cancer (Han D. et al., 2019).

YTHDF1 has been reported to facilitate gastric carcinogenesis by controlling FDZ7 translation (Pi et al., 2020). Herein, we observed the significant upregulation of YTHDF1 in GC tissue samples and its increased expression was associated with poor prognosis, acting as an independent prognostic factor in patients with GC. Knockdown of YTHDF1 repressed GC cell growth and metastasis *in vitro* and *in vivo.* Intersection co-analysis for RNA-seq, Methylated RNA immune-precipitation (MeRIP)-seq/qPCR, Co-immunoprecipitation (Co-IP), RNA immunoprecipitation (RIP)-qPCR, luciferase analysis and RNA pull-down revealed that YTHDF1 facilitated USP14 translation via a m^6^A-dependent way, and USP14 upregulation harbored a positive correlation with YTHDF1 expression and indicated a poor prognosis in GC.

## Materials and Methods

### Clinical Data

The clinichological data of GC patients as well as the relative expression levels of YTHDF1, and proteasomal protein catabolic process-related factors (USP14, ANKIB1, ARIH1, ATXN3, GABARAPL2, NUB1, PRKACA, PSMC1, SIRT2, TLK2, UBQLN1, and WAC) were obtained from Gene Expression Omnibus (GEO) database (*n* = 592, including datasets GSE29272, GSE14210, GSE15459, GSE22377, and GSE51105)^1^ and The Cancer Genome Atlas (TCGA) RNA-seq database (*n* = 292)^2^. The protocols used in our study were approved by the Ethics Committee of the Shanghai Sixth People’s Hospital. The comprehensive clinical and pathological information of GC patients for YTHDF1 expression are summarized in Tables 1, 2 and Supplementary Tables S1, S2.

**TABLE 1:** Correlation of YTHDF1 expression with clinicopathological parameters in GC patients (GSE29272).

Variables	Cases (n)	YTHDF1 expression		*P*
		Low (%)	High (%)	
**Age**				
≤60	75	52 (69.3)	23 (30.7)	0.1675
>60	51	41 (80.4)	10 (19.6)	
**Gender**				
Male	99	75 (75.8)	24 (24.2)	0.3428
Female	27	18 (66.7)	9 (33.3)	
**Tumor grade**				
1∼2	45	32 (71.1)	13 (28.9)	0.609
3	81	61 (75.3)	20 (24.7)	
**Pathological stage**				
I/II	10	10 (100.0)	0 (0.0)	0.0619
III/IV	116	83 (71.6)	33 (28.4)	
**Lymph node metastasis**				
No	26	21 (80.8)	5 (19.2)	0.333
Yes	98	70 (71.4)	28 (28.6)	
Unknown	2	2 (100.0)	0 (0.0)	
**Family history of upper gastrointestinal cancer**				
No	97	69 (71.1)	28 (28.9)	0.2134
Yes	29	24 (82.8)	5 (17.2)	
**Lesion**				
GNCA	71	60 (84.5)	11 (15.5)	0.002**
GCA	55	33 (60.0)	22 (40.0)	

**P < 0.01; GNCA, gastric non-cardia adenocarcinomas; GCA, gastric cardia adenocarcinomas.

**TABLE 2:** Cox regression analysis of YTHDF1 as potential overall survival predictor (GSE29272).

Variables	Univariate cox regression analysis		Multivariate cox regression analysis	
	RR (95% CI)	*P*-value	RR (95% CI)	*P*-value
**Age** (years, ≤ 60 vs. > 60)	0.777 (0.515–1.173)	0.229	NA	NA
**Gender** (Male vs. Female)	1.164 (0.715–1.895)	0.541	NA	NA
**Tumor grade** (I + II vs. III)	0.721 (0.467–1.111)	0.138	NA	NA
**Pathological stage** (I + II vs. III + IV)	0.777 (0.359–1.681)	0.522	NA	NA
**Family history of upper gastrointestinal cancer** (No vs. Yes)	0.831 (0.506–1.364)	0.463	NA	NA
**Lesion** (GNCA vs. GCA)	0.864 (0.572–1.304)	0.486	NA	NA
**Lymph node metastasis** (No vs. Yes)	0.355 (0.197–0.639)	0.001**	0.354 (0.196–0.639)	0.001**
**YTHDF1** (Low vs. High)	0.607 (0.386–0.954)	0.03*	0.607 (0.386–0.955)	0.031*

*P < 0.05; **P < 0.01. RR, relative risk; NA, not analyzed; GNCA, gastric non-cardia adenocarcinomas; GCA, gastric cardia adenocarcinomas.

### Tissue Microarray and Immunohistochemistry Analysis

Human microarrays (TMA) from GC were purchased from Shanghai Outdo Biotech Co. Ltd. (Shanghai, China). TMA1 contained 80 pairs of tumors and matched adjacent tissues (Supplementary Tables S3, S4) and TMA2 included 28 tumors and matched adjacent tissues. Signed written informed consent was obtained and patient’s personal data were deleted. The expression and cellular localization of YTHDF1 and USP14 in GC tissues were detected by immunohistochemistry (IHC) analysis. The GC tissues were stained with anti-YTHDF1 (ab230330, Abcam, United States) and anti-USP14 (ab71165, Abcam, United States) antibodies. The IHC assay involved the following steps: 3 mm thick sections were used in IHC examinations, and were unmasked with sodium citrate-hydrochloric acid buffer (10 mM, pH 6.0) at 90°C for 30 min. Afterward, the sections were incubated with 0.03% hydrogen peroxide for 10 min to eliminate the effect of endogenous peroxidase activity. Next, incubate the sections with blocking serum for 30 min at room temperature. The blocking serum was composed of 0.04% bovine serum albumin (A2153, Sigma- Aldrich, Shanghai, China) and 0.5% normal goat serum (X0907, Dako Corporation, Carpinteria, CA, United States) in phosphate buffered saline (PBS) solution. After dilution of anti-YTHDF1 (1:100, ab230330, Abcam, United States) and anti-USP14 (1:500, ab71165, Abcam, United States) antibodies, they were used for incubation of slides overnight at 4°C. Next, PBS was used to wash sections 3 times for 5 min each. Finally, 0.5% casein and 5% normal serum were added to block non-specific staining for 30 min at room temperature, and the sections were stained with diaminobenzidine substrate and hematoxylin. The tissue slides were evaluated under a microscope (Nikon ECLIPSE Ci) and pictures were scanned by PANNORAMIC MIDI (3DHISTECH, Hungary) and acquired using CaseViewer_2.0 (v2.0.2.61392, 3DHISTECH, Hungary). The histochemistry score as quantification of protein expression was analyzed using DensitoQuant 1.15.4 of QuantCenter (3DHISTECH, Hungary) (Azim et al., 2015; Yeo et al., 2015). IHC scoring was evaluated by two independent clinical pathologists.

### RNA Extraction and Real-Time Quantitative PCR

Total RNA was extracted from GC cell lines using TRIzol reagent (Invitrogen, Thermo Fisher Scientific, United States). The cDNA was synthesized from RNA using the PrimeScriptTM RT kit (RR037A, Takara, Japan). TB Green^TM^ Premix Ex Taq^TM^ II kit (RR820A, Takara, Japan) and QuantStudio 7 Flex were used in real-time quantitative PCR (RT-qPCR) based on the manufacturer’s guidelines. The mRNA expression levels of target genes were measured using the 2^–△^^△^^Ct^ method. β-ACTIN was used as control to normalize mRNA expression. The primers used are listed in Supplementary Table S5.

### Cell Culture

The human GC cell line AGS was purchased from Shanghai Institute of Biochemistry and Cell Biology. Other human GC cell lines (MKN-28, HGC-27, BGC-823, SGC-7901) and the normal human gastric epithelial cell line GES-1 were obtained from stocks at Digestive Disease Laboratory of Shanghai Sixth People’s Hospital. These cell lines were tested to be free of Mycoplasma and were maintained in Ham’s F-12K (Kaighn’s) Medium (Gibco, United States) or Roswell Park Memorial Institute 1640 medium (Hyclone, Logan, Utah) supplemented with 10% heat-inactivated fetal bovine serum (Gibco, United States), 100 μg/mL of streptomycin and 100 U/mL of penicillin (Penicillin-Streptomycin-Glutamine, Gibco, United States) in a humidified incubator containing 5% CO_2_ at a temperature of 37°C. Cells were treated with 100 μM IU1 (Selleck, Houston, TX, United States) wherever mentioned.

### Western Blotting

GC cells were obtained and extracted using Radio Immunoprecipitation Assay Lysis buffer (Beyotime, Shanghai, China) and equal volumes of GC cells extracted solution were separated on 7.5 and 10% SDS-PAGE gels (EpiZyme, Shanghai, China). The protein bands were transferred from SDS-PAGE gels to PVDF membranes (Millipore PVDF 0.45 μm). The primary antibodies anti-YTHDF1 (ab230330, Abcam, United States), anti-USP14 (ab71165, Abcam, United States), and anti-β-actin (Proteintech, IL, United States) were added at a dilution of 1:500 or 1:1,000 according to the manufacturer’s instructions and incubated at 4°C overnight. The secondary antibodies (horseradish peroxidase [HRP] rabbit IgG) were diluted 1:3,000 and incubated with the membranes at room temperature for 2 h. The membranes were washed thrice with Tris-Buffered Saline with Tween-20, and the immunoreactive bands were developed using SuperSignal West Dura Extended Duration Substrate (Thermo Fisher Scientific, IL, United States) based on established protocols. These experiments were repeated more than three times.

### Cell Transfection and Lentiviral Infection

The steps of transient transfection and lentiviral infection were performed as described previously (Liu H. et al., 2018). Plasmid-mediated USP14 (NM_005151) vector, and lentiviral vectors for YTHDF1 knockdown and overexpression (NM_017798) were purchased from Genechem (Shanghai, China) and an empty vector was used as the negative control (NC). The m^6^A binding site mutated USP14 vector was obtained from RiboBio (Guangzhou, China) with mutation from A to C at No.721 sequence site. The additional details relative to overexpression plasmids of USP14 and YTHDF1 are presented in Supplementary Table S6. The target sequence of the two shRNA (shYTHDF1) were 5′-CGAAAGAGTTTGAGTGGAA-3′ and 5′-TCGTTACATCAGAAGGATA-3′ (Supplementary Table S7).

### Cell Proliferation, Migration, and Invasion Assays

Transwell migration and invasion assays were conducted as previously described (Liu H. et al., 2018). Cell proliferation was detected using a CCK-8 assay kit (Dojindo Corp, Japan) and colony formation. These experiments were repeated at least three times. CCK-8 assay: 1 × 10^3^cells were seeded in a 96-well plate. After cells culture for 0, 24, 48, 72, and 96 h, dyeing solution containing 10 μL CCK-8 reagent and 90 μL culture medium were added directly to each well. The cells were then incubated at 37°C for 1 h, and the optional density was measured at 450 nm. Colony formation: GC tumor cells were trypsinized before collection for counting and a total of 1–3 × 10^3^ cells were plated in 6-well plates and cultured at 37°C in an incubator for 7 days. After washing three times using PBS and fixed with 4% paraformaldehyde, colonies were stained by 0.1% crystal violet solution. Cell colonies were then imaged, counted and analyzed.

### Flow-Cytometric Analysis

Cell apoptosis assays and cell cycle assays were performed and three separate experiments were performed for each clone. For cell apoptosis assays: cells were trypsinized and washed with cold PBS (4°C), and then resuspended in Annexin V Binding Buffer in accordance with the manufacturer’s instructions for the APC Annexin V Apoptosis Detection Kit with 7-AAD (Biolegend, San Diego, CA, United States). Five microliter APC Annexin and 7-AAD Viability Staining Solution were added to the cells in the dark conditions, respectively, and incubate at room temperature (25°C) for 15 min. A volume of 400 μL of Annexin V Binding Buffer was then added to each tube before the flow cytometry analysis on a CytoFLEX flow cytometer (Beckman Coulter, CA, United States). Cell apoptosis was analyzed using the CytoFLEX-CytExpert Software 2.4 (Beckman Coulter, CA, United States). For cell cycle assays: after washing cells twice in cold PBS and RNase incubation for 30 min at 37°C, the fixed cells were dyed by PI (Cell Cycle DNA Content Quantitation Assay, Solarbio, Beijing, China) for 30 min at room temperature in the dark. Each sample was then filtered through a 50 μm nylon strainer to obtain single-cell suspension. The samples were then analyzed on CytoFLEX flow cytometer (Beckman Coulter, CA, United States). FlowJo 10.4 software (Becton, Dickinson & Company, NJ, United States) was used for cell cycle analysis.

### Animal Models

The animal experiments were approved by the Ethics Committee of Shanghai Jiao Tong University Affiliated Sixth People’s Hospital. For the subcutaneous xenotransplanted tumor model, BALB/c nude mice (15 mice, 5–6 weeks old) were purchased from JRDUN Biotechnology (Shanghai, China) and fed in specific-pathogen-free animal houses. BGC-823 cells (5 × 10^6^) with shRNAs targeting YTHDF1 (sh-YTHDF1) or shRNAs targeting control (sh-NC) were trypsinized and suspended in 0.1 mL PBS and injected subcutaneously into the BALB/c mice (*n* = 5 mice per group). For the subcutaneous-injected mice, the volume of xenograft growth was examined by measuring tumor width and length every 3 days [volume = (length × width^2^)/2]. After 5 weeks, mice were sacrificed, and the tumor weight was recorded. Tissues were paraffin-embedded, dissected, and stained with hematoxylin-eosin. For the pulmonary metastasis model, mice were randomly divided into 2 groups (*n* = 4 per group) and 2 × 10^6^ cells were injected into the tail vein of the BALB/c nude mice. After 4 weeks, mice were sacrificed and lungs were extracted and fixed 4% paraformaldehyde in PBS. The number of metastatic lung tumors from GC cells was further counted. Tissues were paraffin-embedded, dissected, and stained with hematoxylin-eosin.

### RNA-Seq

Total RNA was isolated from stable YTHDF1 control or knockdown AGS cells using Trizol reagent (Invitrogen) according to the manual’s instruction. Poly(A) mRNA purification was followed and extracted from 50 to 100 ng total RNA using NEB Next^®^ Poly(A) mRNA Magnetic Isolation Module. The library establishment and next generation sequencing (NGS) were performed by Aksomics (Shanghai, China). Every group was sequenced in triplicate.

### RNA Immunoprecipitation (RIP) Assay

RIP assay was carried out by using a RNA Immunoprecipitation Kit (Geneseed Biotech, Guangzhou, China). According to the manufacturer’s instructions, BGC-823 cells were seeded in T75 flasks at 70–80% confluence. 5 μg of YTHDF1 (ab220162, Abcam) antibody and a corresponding control rabbit IgG (2729, Cell Signaling Technology) were conjugated to Dynabeads (11203D, Thermo Fisher Scientific) by incubation for 2 h at 4°C, followed by washing 2 times and incubation with 450 μL supernatants of BGC-823 cells and 350 μL RIP buffer A (provided by RIP Kit) for 2 h at 4°C. After washing with 1 mL RIP buffer B (provided by RIP Kit) for 5 times, beads were resuspended in 300 μL Buffer E (provided by RIP Kit), followed by DNA digestion through DR Columns (provided by RIP Kit). Input and co-immunoprecipitated RNAs were extracted by DR Columns (provided by RIP Kit) and analyzed by RT-qPCR.

### Methylated RNA Immune-Precipitation (MeRIP)-Seq and MeRIP-qPCR

Total RNAs were extracted from sh-YTHDF1 and sh-NC transfected AGS cells by using TRIzol reagent (Invitrogen). Seq-Star^TM^ poly(A) mRNA Isolation Kit (Arraystar, Rockville, United States) was used to obtain complete mRNA. After fragmentating, RNA (100 nucleotides) was incubated with m^6^A antibody (ABE572, Merck Millipore, Germany) for immunoprecipitation based on the instructions of the MeRIP m^6^A Kit (Merck Millipore, Germany). The mRNA with m^6^A enrichment was then assayed using NGS or RT-qPCR. For NGS, RNA fragments were purified from m^6^A-MeRIP and sequenced with Illumina HiSeq X-10 after building the library with the NEBNext Ultra RNA library Prep kit for Illumina (New England BioLabs). The library establishment and NGS were performed by Aksomics (Shanghai, China). MeRIP-qPCR was executed to measure the m^6^A levels of USP14 in GC cells. Primers targeting the m^6^A negative/positive site of MAP2K4 were used as the negative/positive control (Supplementary Table S5).

### Co-immunoprecipitation (Co-IP)

BGC-823 cells were washed with PBS and incubated in 200 μL lysis buffer (50 mM Tris-HCl, pH 7.5, 150 mM NaCl, 15 mM MgCl_2_, 5 mM EDTA, and 0.1% NP-40) containing protease inhibitor Cocktail (Roche, Mannheim, Germany) for subsequent Co-IP. The cell lysates were incubated with specific antibodies against USP14 (ab71165, Abcam, United States) and YTHDF1 (ab220162, Abcam, United States) at 4°C for 2 h, then incubated with 2.5 mg Dynabeads Protein G magnetic beads (Thermo Fisher Scientific, 10004D) at 4°C overnight. The beads were separated and washed with cold PBS, and Western blotting analysis was performed.

### Luciferase Reporter Assay

The fragment of wild-type USP14 CDS (USP14) containing predicted YTHDF1 target sites was amplified by PCR and cloned into pGL3 vector (Promega, United States), which included Firefly luciferase reporter genes. The wild-type YTHDF1(YTHDF1-WT) was amplified by PCR and cloned into pcDNA3.1 vector. The mutant YTHDF1 (YTHDF1-MUT) was then generated by mutating the YTH domain of YTHDF1 using Gene Mutation Kit (Takara, JAPAN). Next, the YTHDF1-MUT or YTHDF1-WT plasmid were co-transfected with USP14 and pRL-TK vector which included Renilla luciferase reporter genes (Promega, United States) into AGS cells. The pcDNA3.1 vector was used as negative control. After 48 h, the cells were harvested, and the Firefly and Renilla luciferase activities were measured based on a dual-luciferase reporter assay system (Promega, United States). The relative luciferase activity was normalized to Renilla luciferase activity (F-Luc/R-Luc).

### Biotin-Coupled Probe RNA Pull Down Assay

To confirm whether YTHDF1 protein could be pulled down from USP14 mRNA in GC cells, biotinylated-USP14 probe was synthesized by RiboBio (Guangzhou, China) (Supplementary Table S8). BGC-823 cells (1 × 10^7^) were lysed and incubated with biotin-labeled USP14 probe. Next, the biotin-coupled RNA complex was pulled down using streptavidin-coated magnetic beads adsorption. The enriched YTHDF1 was analyzed by western blot analysis.

### Statistical Analysis

Each experiment was repeated at least 3 times. Statistical tests were performed using SPSS 22.0 (IBM, SPSS, Chicago, IL, United States) and GraphPad version 8.0 Prism (GraphPad Software, La Jolla, CA, United States). The continuous variables were described as the mean ± standard deviations. Significant differences were evaluated via Student’s unpaired *t*-test or the chi-square test for comparisons of two groups. For multiple groups, significant differences were analyzed by one-way ANOVA (followed by Tukey’s honest test). Pearson’s correlation coefficient analysis was used for analyzing correlations. Survival curves were generated using the Kaplan-Meier method and log-rank testing. A *p*-value less than 0.05 was considered statistically significant.

## Results

### High Expression of YTHDF1 Indicated Poor Prognosis in Patients With GC

**FIGURE 1:**
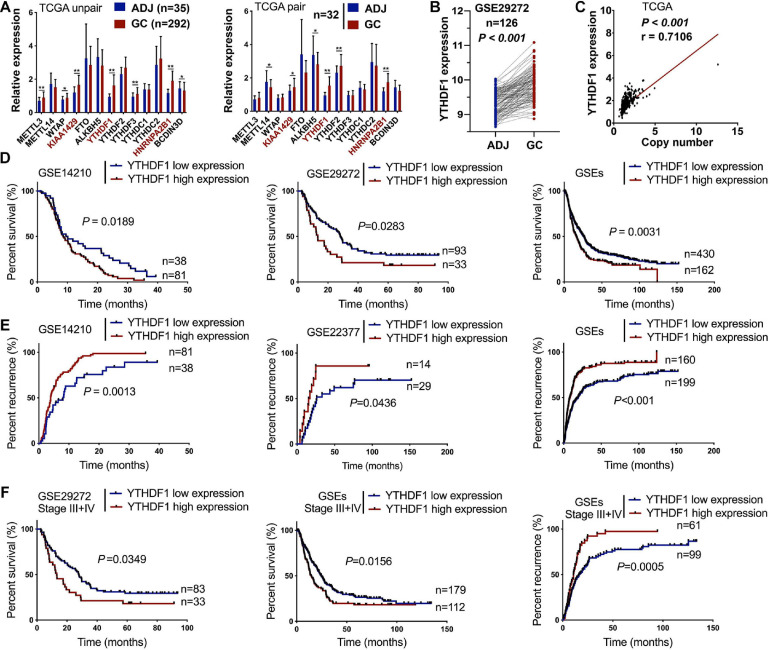
High expression of YTHDF1 predicts a poor prognosis in patients with GC. **(A)** Gene expression of m^6^A-related compositions in patients with GC according to TCGA dataset (unpaired or paired). **(B)** Gene expression of YTHDF1 in patients with GC according to GSE29272 dataset (*n* = 126). **(C)** Correlation analysis between gene expression and the copy number status of YTHDF1 in TCGA-GC dataset. **(D,E)** Kaplan–Meier analysis of GC patients in GSEs dataset for the correlations between YTHDF1 expression and overall survival. as well as tumor recurrence. **(F)** Kaplan–Meier analysis of GC patients with Stage III and Stage IV in GSEs database. ADJ, adjacent normal; GC, gastric cancer. Data are shown as means ± S.D. *P < 0.05, **P < 0.01.

Recent studies have revealed m^6^A methylation components can act as tumor-associated factors, including “writers” (METTL3/14, WTAP and KIAA1429), “erasers” (FTO and ALKBH5), and “readers” (YTHDF1/2/3, HNRNPA2B1, BCDIN3D) (Lin et al., 2016; Ma et al., 2017; Liu J. et al., 2018; Chen et al., 2019). We herein measured the expression of these m^6^A-related members in 418 patients with GC from TCGA (*n* = 292) and GSE29272 (*n* = 126) databases (clinicopathological data shown in Tables 1, 2 and Supplementary Tables S1, S2). Compared with the adjacent normal tissues, KIAA1429, YTHDF1, and HNRNPA2B1 were upregulated in GC tissues from TCGA (Figure 1A). Furthermore, YTHDF1 expression was significantly increased both in gastric cardia adenocarcinoma (GCA) and gastric non-cardia adenocarcinoma (GNCA) according to the GSE29272 dataset compared with their pair-matched normal tissues (Figure 1B and Supplementary Figure S1A). The genetic alterations and copy numbers of *YTHDF1* were analyzed using the cBioPortal (cBio Cancer Genomics Portal^3^) (Cerami et al., 2012; Gao et al., 2013), which indicated that the *YTHDF1* gene was frequently amplified and the mRNA expression of *YTHDF1* had a positive correlation with its copy number in TCGA cohort (*P* < 0.001, *r* = 0.7106, Figure 1C). The *YTHDF1* gene was altered in 12.09% of 612 cases, including mutation (3 cases, 4.05%), amplification (59 cases, 79.73%), and multiple alterations (1 case, 1.35%) (Supplementary Figure S1B).

Furthermore, Kaplan–Meier survival analysis demonstrated that GC patients with high YTHDF1 expression possessed poorer overall survival (*P* = 0.0352, Supplementary Figure S1C). Consistently, the cases with YTHDF1-high expression displayed poor survival (Figure 1D) and tumor recurrence (Figure 1E) as compared with those with low expression in GEO database. Although *YTHDF1* expression showed no significant differences at different stages both in TCGA and the GSE29272 dataset, GC patients in late stages (FIGO stage III or IV) rather than in early stages (FIGO stage I or II) with high *YTHDF1* expression tended to exhibit poor survival and tumor recurrence (Figure 1F and Supplementary Figures S2A–C). Therefore, these results indicated that YTHDF1 as a m^6^A reader was upregulated in GC tissues and was involved with a poor prognosis in GC patients.

Receiver operating characteristic (ROC) curve analysis was performed to determine the cutoff values of several m^6^A-related proteins (METTL3/14, YTHDF1/2, ALKBH5, FTO, and HNRNPA2B1) in TCGA cohort (Supplementary Figure S1C). The area under the ROC curve (AUC), cutoff value, sensitivity, and specificity of YTHDF1 were 0.52, 10.16, 97.9, and 8.2%, respectively, in TCGA cohort (Supplementary Figure S1C), indicating that YTHDF1 was a promising clinical marker in GC patients. Up-expression of YTHDF1 was significantly associated with N stage (TCGA cohort, *P* = 0.019), cancer lesion (GEO cohort, *P* = 0.002), and tumor size (TMA1, *P* = 0.015) in patients with GC (Table 1 and Supplementary Tables S1, S3). Moreover, univariate and multivariate Cox regression analyses suggested that YTHDF1 expression and lymph node metastasis were independent prognostic factors of poor survival in patients with GC from the GSE29272 dataset, while pathological stage and age were independent prognostic factors in TCGA cohort (Table 2 and Supplementary Tables S2, S4).

### YTHDF1 Knockdown Repressed Proliferation and Invasion *in vivo* and *in vitro*

**FIGURE 2:**
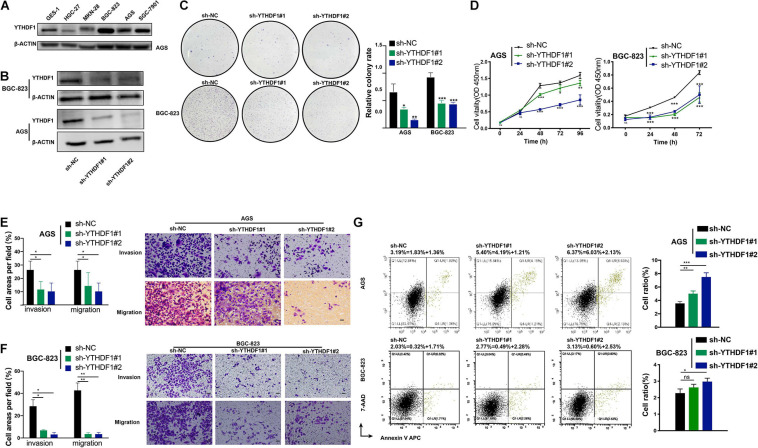
Inhibition of YTHDF1 suppresses cell growth, migration and invasion *in vitro*. **(A)** Western blot analysis for YTHDF1 expression in GES-1 and GC cell lines (AGS, BGC-823, SGC-7901, MKN-28, and HGC-27). **(B)** Western blot analysis for YTHDF1 expression in AGS and BGC-823 cells infected with two independent shRNAs targeting YTHDF1 (sh-YTHDF1#1/2) or a control shRNA (sh-NC). **(C)** Colony formation assays of AGS and BGC-823 cells described in **(B)**. **(D)** CCK8 assays were performed to determine cell growth in YTHDF1 deficient AGS and BGC-823 cells as described in **(B)**. **(E,F)** Knockdown of YTHDF1 decreased the abilities of migration and invasion of AGS and BGC-823 cells. Scale bar, 50 μm. **(G)** Cell apoptosis analysis was used to compare down-expression of YTHDF1 (sh-YTHDF1#1/2) with the sh-NC group in BGC-823 and AGS cells. *P < 0.05, **P < 0.01, ***P < 0.001; ns, no significance.

We analyzed the RNA expression level of *YTHDF1* using the Cancer Cell Line Encyclopedia^4^ database, in which AGS had the highest expression of YTHDF1 among GC cell lines (Supplementary Figure S3A). YTHDF1 protein expression was measured in various GC cell lines by western blotting analysis, which indicated that it was much higher in BGC-823, AGS, SGC-7901 cell lines than the normal gastric gland cell line GES-1 (Figure 2A). We then characterized the altered cellular phenotypes by establishing stable shRNA-expressing AGS and BGC-823 cell lines (Figure 2B). YTHDF1 silencing significantly impaired cell growth of AGS and BGC-823 cells as indicated by CCK-8 assays and colony formation assays (Figures 2C,D) and repressed cell migration and invasion abilities as indicated by the Transwell assay (Figures 2E,F). Flow-cytometric analysis showed that downregulation of YTHDF1 induced cell cycle arrest at the S phase (Supplementary Figure S3B) and cell apoptosis (Figure 2G and Supplementary Figure S3C) as compared with the sh-NC group in BGC-823 and AGS cells.

**FIGURE 3:**
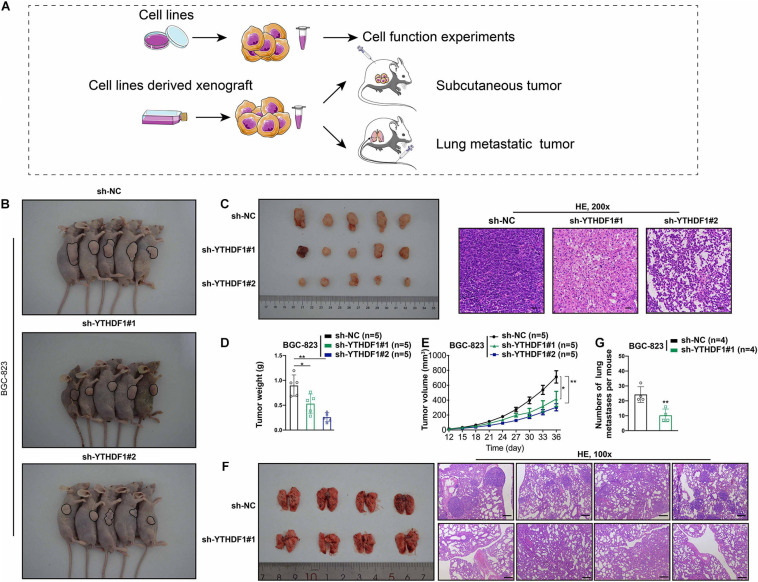
YTHDF1 deficiency inhibits tumor growth and metastasis *in vivo*. **(A)** Different systems were applied including GC cell lines and cell line-derived xenograft. **(B)** Images of the sacrificed nude mice injected with YTHDF1 deficient BGC-823 cells and its control group in mice subcutaneous tumor model (*n* = 5). **(C)** (Left) Tumors were dissected from the nude mice of each group and photographed at 36 days after transplantation. (Right) Representative histology images in mice subcutaneous tumor model (*n* = 5, 200×). Scale bar, 50 μm. **(D,E)** Tumor weight and the tumor growth curve were measured in YTHDF1 down-expressed cells and its control group. **(F)** (Left) Images of pulmonary metastases in mice lung metastasis model (*n* = 4). Black arrows: metastatic nodules. (Right) Representative histology images in mice lung metastasis tumor model (*n* = 4, 100×). Scale bar, 200 μm. **(G)** The numbers of lung metastatic nodules in YTHDF1 down-expressed cells and its control group. GC, gastric cancer. Data are shown as means ± S.D. *P < 0.05, **P < 0.01.

The oncogenic role of YTHDF1 in GC tumorigenesis was investigated by subcutaneous or tail vein injection with control cells or YTHDF1-deficient BGC-823 cells into immunocompromised nude mice (Figure 3A). Low YTHDF1 expression lead to delayed cell growth of BGC-823-engrafted tumors (Figures 3B,C), and the tumor weight and volume in YTHDF1 deficient tumors were reduced in comparison with the sh-NC group (Figures 3D,E). The effects of YTHDF1 knockdown on GC with lung metastasis were observed and the number of metastatic lung tumors from GC was markedly decreased in YTHDF1-deficient mice as compared with the sh-NC group (Figures 3F,G).

### Transcriptome-Wide Identification of YTHDF1 Targets in GC Cells

**FIGURE 4:**
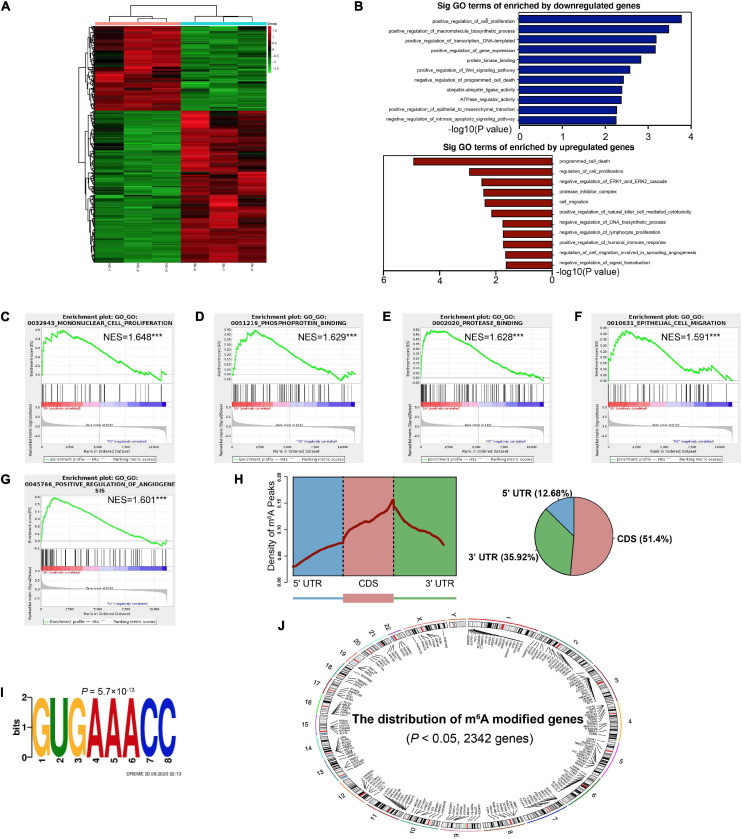
Identification of the YTHDF1 targets in GC cells. **(A)** Heatmap of DEGs identified by RNA-seq. **(B)** GO enrichment analysis of DEGs. **(C–G)** GSEA plots showing the pathways of DEGs enriched by YTHDF1 were involved in GC cells. **(H)** Metagene profiles of m^6^A enrichment across mRNA transcriptome in BGC-823 cells. **(I)** The m^6^A motif detected by the DREME motif analysis with m^6^A-seq results. **(J)** The distribution of m^6^A peaks on different chromosomes. DEGs, differentially expressed genes; GC, gastric cancer; GO, Gene ontology; GSEA, gene set enrichment analysis. ***P < 0.001.

In order to comprehensively discover the downstream mechanisms of YTHDF1 in GC tumorigenesis, we performed a RNA-seq analysis, which revealed obvious subsets of transcripts that were altered due to the downregulation of YTHDF1 in AGS cells (*P* < 0.05, Supplementary Table S9). Among these, 430 genes were altered with absolute value of log_2_(Fold Change) > 0.585, including 276 upregulated and 154 downregulated genes (Figure 4A and Supplementary Figures S4A,B). These altered genes were distributed in different position across chromosomes (Supplementary Figure S4C). Gene ontology (GO) analysis and gene set enrichment analysis (GSEA) were used to analyze the regulatory roles of YTHDF1 in GC. GO analysis revealed that the differentially-expressed genes (DEGs) regulated by YTHDF1 participated in cell proliferation, protease inhibitor complex activity, cell migration, the Wnt signaling pathway, programmed cell death, ubiquitin-ubiquitin ligase activity and the ERK1 and ERK2 cascade (Figure 4B). GSEA analysis also indicated that YTHDF1 upregulated or downregulated target genes were related to mononuclear cell proliferation, phosphoprotein binding, protease binding, and epithelial cell migration (Figures 4C–G and Supplementary Figure S4D), suggesting that YTHDF1 could act as an oncogene in GC.

YTHDF1 can promote protein translation in a m^6^A-dependent manner (Wang et al., 2015). We then identified possible transcripts modified by m^6^A methylation in sh-YTHDF1 and sh-NC transfected AGS cells. Using m^6^A-seq analysis, we identified 6753 m^6^A peaks with significant dysregulation (*P* < 0.05, Supplementary Table S10). The m^6^A peaks were uniquely detected with the GUGAAACC motifs (*P* = 5.7 × 10^–13^), mainly enriched in 3′UTR regions (Figures 4H,I), and were located on different chromosomes (Figure 4J). Functional annotation of these m^6^A-modified mRNAs suggested several obvious Kyoto Encyclopedia of Genes and Genomes (KEGG) gene clusters such as the Wnt signaling and MAPK signaling pathways (Supplementary Table S11).

### USP14 Was a m^6^A Modification Target of YTHDF1 in GC Cells

**FIGURE 5:**
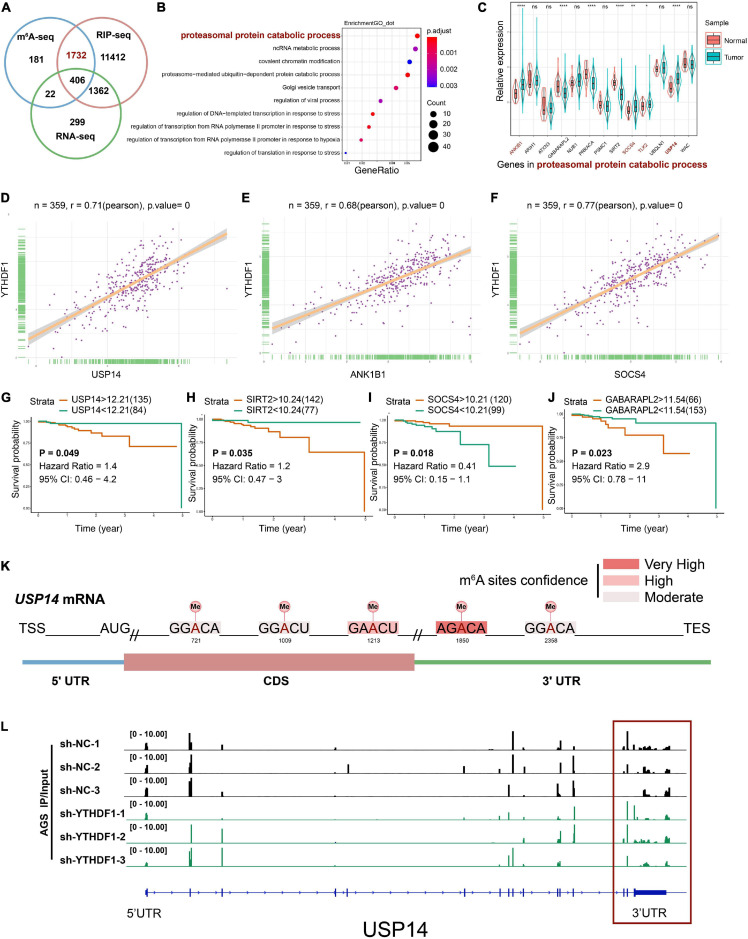
USP14 as the m^6^A modification target of YTHDF1. **(A)** Overlapping analysis of genes identified by MeRIP-seq, RIP-seq, and RNA-seq. **(B)** GO analysis of genes described in **(A)**. **(C)** The expression of genes related to proteasomal protein catabolic process in patients with GC according to TCGA dataset. **(D–F)** Correlation analysis between YTHDF1 expression and USP14, ANK1B1 and SOCS4 expression in TCGA-GC dataset. **(G–J)** Kaplan-Meier analysis of GC patients in TCGA dataset for the correlations between USP14/SIRT2/SOCS4/GABARAPL2 expression and overall survival. **(K)** Predicted m^6^A sites in USP14 mRNA by SRAMP program, especially in 3’UTR. **(L)** The m^6^A abundance on USP14 mRNA transcripts in sh-YTHDF1 and sh-NC infected AGS cells as examined by MeRIP-seq. Data represented the mean ± SD. GC, gastric cancer; GO, Gene ontology; TCGA, The Cancer Genome Atlas; MeRIP, Methylated RNA immune-precipitation. Data are shown as means. *P < 0.05, **P < 0.01, ****P < 0.0001; ns, no significance.

RIP-seq has been used to screen YTHDF1-specific binding genes in Hela cells and 14,912 potential genes were found to bind with YTHDF1 (Wang et al., 2015). To explore the direct interactions between YTHDF1 and its targeted transcripts, the intersection co-analysis of RNA-seq, MeRIP-seq, and RIP-seq suggested that 2138 YTHDF1-binding genes were marked by m^6^A, among which 1,732 (81.01%) genes were not altered under YTHDF1 deficiency as shown by RNA-seq (Figure 5A). These results demonstrated that YTHDF1 did not affect the RNA abundance of these 1732 targets in GC cells, which is in accordance with previous findings that YTHDF1 regulates protein synthesis in a m^6^A-dependent manner in ovarian cancer (Liu T. et al., 2020). In addition, functional annotation confirmed the foregoing 1,732 genes were involved in multiple biological processes covering proteasomal protein catabolic processes (Figure 5B). The expression of these proteasomal protein catabolic process-related factors was analyzed in patients with GC according to TCGA dataset (Figure 5C). YTHDF1 exhibited a positive correlation with proteasomal protein catabolic process-related factors in GC tissue samples from TCGA cohort (Figures 5D–F and Supplementary Figure S5A), but showed an opposite tendency between gene expression and tumor prognosis, with only ubiquitin-specific protease 14 (USP14) upregulation indicating a shorter overall survival in patients with GC (Figures 5G–J and Supplementary Figure S5B). RNA m^6^A enrichment tended to locate at the 3’-UTR and near the stop codons of mRNA which contains the classic RRACH sequence (R = G/A and H = A/C/U) (Meyer et al., 2012; Yi et al., 2020). Analysis of USP14 mRNA with SRAMP program (Zhou et al., 2016) predicted the potential m^6^A sites on 3’UTR (Figure 5K and Supplementary Table S12), which was consistent with our results showing m^6^A peaks were located on USP14 mRNA in sh-YTHDF1 and sh-NC transfected AGS cells, especially on the 3’UTR as demonstrated by the MeRIP-seq findings (Figure 5L and Supplementary Figure S5C). USP14 as a component of proteasome-associated deubiquitinating enzyme complex can eliminate ubiquitins from proteasome-bound substrates and inhibit the proteasome non-catalytically (Chen et al., 2018). Consistently, StarBase2.0 predicted that USP14 mRNA can be enriched by YTHDF1 (ENCORI: The Encyclopedia of RNA Interactomes) (Li et al., 2014). These data suggested that USP14 was a m^6^A modification target of YTHDF1 in GC cells.

### Upregulation of USP14 Reversed the Tumor Depressed Phenotype in YTHDF1-Knochdown GC Cells

**FIGURE 6:**
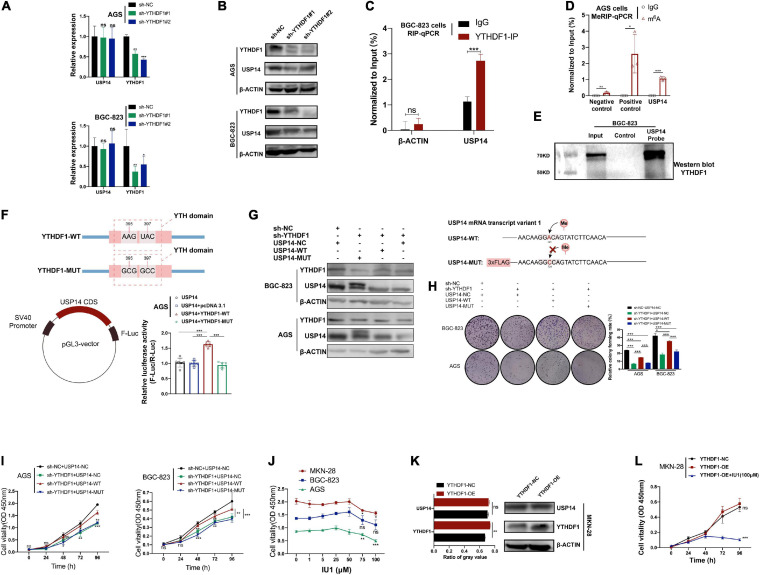
USP14 rescues the tumor suppressive effect caused by YTHDF1 deficiency. **(A)** RT-qPCR analysis of the relative RNA level of USP14 in sh-YTHDF1 and sh-NC infected AGS and BGC-823 cells. **(B)** Western blot analysis of the protein level of USP14 in sh-YTHDF1 and sh-NC infected AGS and BGC-823 cells. **(C)** YTHDF1-RIP-qPCR confirmed the interrelationship between YTHDF1 and USP14 mRNA in BGC-823 cells. **(D)** MeRIP-qPCR validation of m^6^A levels of USP14 in AGS cells. Primers to m^6^A negative region of MAP2K4 as the negative control and primers to m^6^A positive region of MAP2K4 as the positive control. **(E)** RNA pull-down with a biotin-labeled USP14 probe was implemented in BGC-823 cells, followed by western blot to test the enrichment of YTHDF1. **(F)** (up) Diagrammatic sketch showed the construction of YTHDF1-WT and YTHDF1-MUT. (down-left) Diagrammatic sketch showed that the fragment of wild-type USP14 CDS (USP14) containing predicted YTHDF1 target sites was cloned into pGL3 vector with Firefly luciferase reporter genes. (down-right) The luciferase activity analysis in AGS cells. **(G)** (Left) Western blot analysis of the protein levels of YTHDF1 and USP14 in YTHDF1-deficient AGS and BGC-823 cells under overexpression of USP14 (USP14-WT) or potential m^6^A binding site mutated USP14 (USP14-MUT). (Right) Diagrammatic sketch showed the construction of USP14-WT and USP14-MUT. **(H,I)** Cell growth was measured by colony formation and CCK8 assays in AGS and BGC-823 cells described in **(G)**. **(J)** Different dosages of IU1 (0, 1, 5, 25, 50, 75, and 100 μM) were used in MKN-28, BGC-823 and AGS GC cell lines. **(K)** Western blot analysis and gray value analysis of the protein level of USP14 and YTHDF1 in YTHDF1-overexpressed and normal control MKN-28 cells. **(L)** Cell growth was measured by CCK8 assays in MKN-28 cells described in **(K)**. GC, gastric cancer; MeRIP, Methylated RNA immune-precipitation. Data represented the mean ± SD. *P < 0.05, **P < 0.01, ***P < 0.001; ns, no significance.

In order to confirm whether YTHDF1 could affect USP14 protein translation in GC cells, we measured the effects of YTHDF1 on transcription and translation levels of USP14. We found that knockdown of YTHDF1 decreased the protein abundance of USP14 rather than its transcription levels in AGS and BGC-823 cells as expected by RT-qPCR and western blotting analysis (Figures 6A,B). Based on the Co-IP assay, YTHDF1 protein was not bound to USP14 protein in BGC-823 cells (Supplementary Figure S6A). Instead, RIP-qPCR of YTHDF1 was used to certify the interrelationship between YTHDF1 and USP14 mRNA and we found that USP14 RNA was enriched in YTHDF1 bound RNA in BGC-823 GC cells (Figure 6C). To further explore the effects of m^6^A modification on the status of USP14 mRNA, we used MeRIP-qPCR to indicate significant m^6^A enrichment sites in USP14 mRNA as compared with the control group in AGS cells (Figure 6D). To confirm the binding of USP14 mRNA with YTHDF1 protein, a RNA pull down assay was performed in BGC-823 cells using a biotinylated USP14 probe and showed that YTHDF1 was abundantly pulled down by the biotin-coupled USP14 probe rather than the control probe in BGC-823 cells (Figure 6E). Additionally, we intended to construct a pGL3-USP14 vector with the USP14 CDS cloned into pGL3 vector with Firefly luciferase reporter genes. The luciferase assay showed that YTHDF1-WT rather than YTHDF1-MUT enhanced expression of USP14 in AGS cells (Figure 6F). These data illustrated that YTHDF1 could directly bind with USP14 mRNA and facilitated its protein translation in a m^6^A-dependent manner.

According to a previous study, silencing of USP14 induced GC cell apoptosis (Fu et al., 2018). We then investigated whether overexpression of USP14 could restore the delayed tumor progression phenotype in YTHDF1-deficient AGS and BGC-823 cells. The overexpression of USP14 in these two cell lines was determined by western blotting analysis (Supplementary Figure S6B). Knockdown of YTHDF1 suppressed cell growth and colony formation, while overexpression of USP14 could rescue sh-YTHDF1 expressing GC cells from these effects (Supplementary Figures S6C,D). Furthermore, according to SRAMP website prediction of m^6^A site on USP14 mRNA transcript variant 1 and the primer used in MeRIP-qPCR, we constructed the USP14- MUT vector linked with 3×FLAG (Figure 6G). Because of USP14-MUT vector with 3×FLAG (∼2 kDa), two protein bands were endogenous USP14 and overexpressed USP14 protein with 3×FLAG, respectively (Figure 6G). We then used this potential m^6^A binding site mutated USP14 in the same experiments, which could not rescue the impaired effects caused by sh-YTHDF1 (Figures 6G–I). In addition, IU1, a small-molecule inhibitor of USP14, accelerated the degradation of a subset of proteasome substrates and suppressed cell proliferation, migration, and invasion in lung cancer and cervical cancer (Lee et al., 2010; Han K.H. et al., 2019; Xu et al., 2020). The IC_50_ of IU1 is 4.7 μM^5^, and concentrations of 50–100 μM IU1 have been used in multiple cancer cells (Li H. et al., 2019; Xia et al., 2019; Sharma et al., 2020; Sharma and Almasan, 2020). We found that the viability of AGS and MKN-28 cells was remarkably suppressed by 75–100 μM IU1, while not in BGC-823 cells (Figure 6J). The cell migration and invasion induced by YTHDF1 overexpression were reversed by exposure to IU1 (100 μM) (Figures 6K,L and Supplementary Figure S6E).

### YTHDF1 Harbored a Positive Correlation With USP14 Expression in GC Patients

**FIGURE 7:**
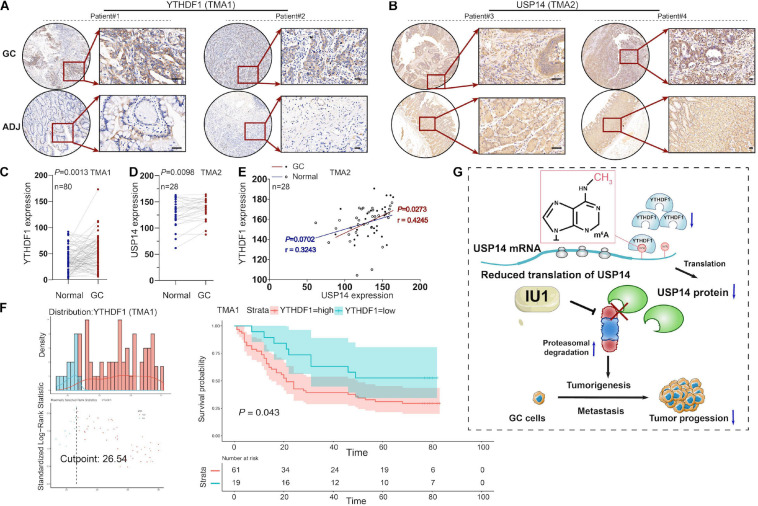
The correlation between YTHDF1 and USP14 expression in GC patients. **(A)** Representative immunohistochemical images of YTHDF1 expression in primary gastric tumor tissues and normal gastric gland. Scale bar, 50 μm. **(B)** Relative USP14 protein expression in normal gastric gland and GC specimens assessed by immunohistochemistry in TMA2 (*n* = 28). Scale bar, 50 μm. **(C)** Comparison between protein expression of YTHDF1 in patients with GC and their adjacent normal tissues according to TMA1 dataset (*n* = 80). **(D)** Comparison between protein expression of USP14 in patients with GC and their adjacent normal tissues according to TMA2 dataset (*n* = 28). **(E)** The correlation analysis of the protein expressions of YTHDF1 and USP14 in GC samples and their adjacent normal tissues (*n* = 28; Pearson’s and spearman’s correlation test). **(F)** Kaplan-Meier analysis of GC patients in TMA1 dataset (*n* = 80) for the correlations between YTHDF1 expression and overall survival. **(G)** Proposed model underlying the roles of YTHDF1-mediated USP14 translation in GC. GC, gastric cancer; TMA, tissue microarray; Data are shown as means ± S.D.

To further investigate the expression of YTHDF1 or USP14 in GC patients, IHC analysis was performed within the TMA1 and TMA2 cohorts containing 80 and 28 pairs of GC samples, respectively (Figures 7A–F). We found that YTHDF1 expression was markedly elevated in GC tissues as compared with the normal tissues preferentially in the TMA1 cohort (Figures 7A,C) rather than in the TMA2 cohort (Supplementary Figures S6F,G), while USP14 was upregulated in GC tissues in the TMA2 cohort (Figures 7B,D). Pearson correlation analysis demonstrated that YTHDF1 harbored a positive correlation with USP14 expression in GC tissues in the TMA2 cohort (*P* = 0.0273, Figure 7E). Survival analysis suggested GC patients with high-regulated YTHDF1 (TMA1 cohort) or USP14 (TCGA cohort) expression exhibited worse overall survival as compared with patients having low YTHDF1 or USP14 expression (Figures 5G, 7F).

## Discussion

Increasing studies have revealed that m^6^A modification plays a role in both physio-biochemical and pathophysiological processes, including carcinogenesis (Cheng et al., 2019; Jin et al., 2019; Li T. et al., 2019). Tumor growth and invasion can be effectively controlled by m^6^A-related “writers,” “readers,” and “erasers,” which determine the fate of m^6^A-modified mRNAs (Lin et al., 2016; Ma et al., 2017; Li N. et al., 2020; Wang et al., 2020). Higher expression of YTHDF1 is related to worse prognosis in patients with hepatocellular carcinoma (Liu X. et al., 2020). We herein found that YTHDF1 as a m^6^A “reader” was upregulated in GC tissues. About 93.8% of GC patients (Supplementary Figure S1C) exhibited relatively increased YTHDF1 expression, and its upregulation predicted a poor prognosis in patients with GC. A shortcoming of our clinical evidence is that we mainly use public database and the sample size of TMA is limited. However, these findings illuminate us that the m^6^A might take part in GC tumorigenesis to a certain degree. In addition, YTHDF1 deficiency impaired GC cell growth and metastasis and induced cell apoptosis both *in vitro* and *in vivo*. The previous experiments indicated YTHDF1 likely represented a common oncogenic driver in gastric carcinogenesis.

RNA-seq and GO analyses indicated that YTHDF1-regulated DEGs were enriched in cell proliferation, migration and metastatic signaling pathways. Intersection co-analysis for RNA-seq, MeRIP-seq and RIP-seq revealed that YTHDF1 could mediate m^6^A methylation to affect proteasomal protein catabolic process, of which USP14 acted as an important target of YTHDF1. The inhibition of USP14 can be speculated to be particularly cytotoxic to cancer cells as it could block proteasome function and increase proteasomal substrates (D’Arcy et al., 2015). Moreover, USP14 is associated with tumorigenesis and drug resistance including breast cancer, lung cancer, and GC (Fu et al., 2018; Han K.H. et al., 2019; Xia et al., 2019). Recently, researches have revealed USP14 enhances cisplatin resistance through affecting Akt/ERK signaling pathways and accelerates cell proliferation and migration in GC (Fu et al., 2018; Han K.H. et al., 2019; Xia et al., 2019). We herein found that USP14 was identified as a downstream target of YTHDF1, and had a positive correlation with YTHDF1 expression, which indicated poor prognosis in GC.

It has been demonstrated that inhibition of USP14-mediated androgen receptor (AR) deubiquitination contributes to the downregulation of AR proteins and suppression of AR-related signaling pathways, such as Wnt/β-catenin in breast cancer (Liao et al., 2018; Xia et al., 2019). YTHDF1 can promote gastric carcinogenesis by controlling translation of Frizzled7 in GC (Pi et al., 2020). We herein found that YTHDF1 could promote USP14 protein translation in a m^6^A-dependent manner and USP14 overexpression reversed the tumor suppressive effects caused by YTHDF1 knockdown in GC cells.

In addition, the regulating or inhibiting factors of m^6^A modifications may act as potential strategies for cancer treatments, as observed for MA2 in glioblastoma multiforme, R-2HG/SPI1/FB23-2 in acute myeloid leukemia, and CA4 in colorectal cancer (Zhang et al., 2016; Weng et al., 2017; Su et al., 2018; Huang et al., 2019). We herein found that IU1 as an inhibitor of USP14 could repress cell growth and restore the tumor-promoting effects induced by YTHDF1 in GC cells. Our findings indicated that YTHDF1 could promote USP14 protein translation in a m^6^A-dependent manner, leading to gastric carcinogenesis (Figure 7G).

YTHDF1 can promote GC progression via affecting translation of Frizzled7 in GC, which is an important receptor to transmit Wnt signaling (Pi et al., 2020). To our knowledge, we provides evidence that m^6^A RNA methylation can modulate not only the Wnt signaling but also the ubiquitin-related pathway to influence GC progression. Since the dysregulation of USP14 has been implicated in multiple human cancers (Li H. et al., 2019; Sharma et al., 2020), USP14 inhibitors may be also applicable to treat other cancers with altered USP14 activity. In conclusion, our results prove that YTHDF1 recognizes the m^6^A target on USP14 mRNA and subsequently promotes the translation of USP14. Based on our findings and the above research reports, we believe that the YTHDF1-USP14 expression mechanism has important significance in GC development, and should be forward investigated for GC prognosis, treatment or diagnosis.

## Data Availability Statement

The datasets presented in this study can be found in online repositories. The names of the repository and accession number(s) can be found below: GSE166972. The information can be found in the article/Supplementary Material.

## Ethics Statement

The studies involving human participants were reviewed and approved by the Ethics Committee of the Shanghai Sixth People’s Hospital. The patients/participants provided their written informed consent to participate in this study. The animal study was reviewed and approved by the Ethics Committee of Shanghai Jiao Tong University Affiliated Sixth People’s Hospital.

## Author Contributions

JZ and J-SZ designed this study. X-YC drafted the manuscript and conducted the statistical analysis. X-YC and RL performed the experiments. Y-CY, H-NF, and MC collected the data. JZ revised this manuscript. All authors read and approved the final manuscript.

## Conflict of Interest

The authors declare that the research was conducted in the absence of any commercial or financial relationships that could be construed as a potential conflict of interest.

## Footnotes

1 http://kmplot.com/analysis/

2 https://genome-cancer.ucsc.edu

3 http://www.cbioportal.org

4 https://portals.broadinstitute.org/ccle

5 https://www.selleck.cn/products/iu1.html

## Abbreviations

3′-UTR: 3′-prime untranslated region
AR: androgen receptor
DEGs: differentially expressed genes
Co-IP: Co-immunoprecipitation
GC: gastric cancer
GEO: Gene Expression Omnibus
GO: Gene ontology
GSEA: gene set enrichment analysis
IHC: immunohistochemistry
KEGG: Kyoto Encyclopedia of Genes and Genomes
m^6^A: N6-methyladenosine
MeRIP: Magna methylated RNA immune-precipitation
NC: negative control
PBS: phosphate buffered saline
RT-qPCR: quantitative real-time PCR
RIP: RNA immunoprecipitation
TCGA: The Cancer Genome Atlas
TMA: Tissue microarray
USP14: Ubiquitin-specific protease 14.
