# Supplementary material for: The m6A Reader YTHDF1 Facilitates the Tumorigenesis and Metastasis of Gastric Cancer via USP14 Translation in an m6A-Dependent Manner
Source: Front Cell Dev Biol. 2021 Mar 15;9:647702. doi: 10.3389/fcell.2021.647702 (PMC8006284; doi:10.3389/fcell.2021.647702)
Supplement: Supplementary file 2 [file Data_Sheet_1.pdf]

Supplementary Figure Legends

Figure S1, related to Figure 1. (A) Heatmap of m^6A-related gene expression in patients with GC according to GSE29272 dataset. (B) Gene mutation rates of YTHDF1 gene in GC according to cBioPortal datasets. (C) ROC and Kaplan–Meier analysis of GC patients in TCGA database for the correlations between m^6A-related genes expression and disease-free survival as well as overall survival. GC, gastric cancer.

Figure S2, related to Figure 1. (A and B) Comparison the relative YTHDF1 expression between FIGO stage I+II and III+IV. (C) Kaplan–Meier analysis of GC patients at different FIGO stages in GSEs database. GC, gastric cancer.

Figure S3, related to Figure 2. (A) Relative expression of YTHDF1 in 37 GC cell lines according to CCLE dataset. (B) Cell cycle analysis was applied to compare sh-YTHDF1 with the sh-NC group in BGC-823 and AGS cells. (C) Early and late apoptosis in BGC-823 and AGS cells. GC, gastric cancer; CCLE, Cancer Cell Line Encyclopedia; Data are shown as means ± S.D. ns, no significance; **P*<0.05, ***P*<0.01, ****P*<0.001;

Figure S4, related to Figure 4. (A and B) PCA and volcano plot were used to describe the DEGs identified by RNA-seq. (C) The distribution of DEGs on different chromosomes. (D) GSEA plots showing the pathways of other DEGs enriched by YTHDF1 in AGS cells. DEGs, differentially-expressed genes. ****P*<0.001.

Figure S5, related to Figure 5. (A) Correlation analysis between YTHDF1 expression and expression of other genes from proteasomal protein catabolic process in TCGA GC dataset. (B) Kaplan-Meier analysis of GC patients in TCGA dataset for the correlations between expression of other genes from proteasomal protein catabolic process and overall survival. (C) The m^6A abundance on USP14 mRNA transcripts with separate IP track and input track in sh-YTHDF1 and sh-NC infected AGS cells as examined by MeRIP-seq.

Figure S6, related to Figure 6 and Figure7. (A) Co-IP of USP14 and YTHDF1 in BGC-823 cells. (B) Western blot analysis of the protein levels of YTHDF1 and USP14 in YTHDF1-deficient AGS and BGC-823 cells under overexpression of USP14. (C-D) Cell growth was measured by colony formation and CCK8 assays in AGS and BGC-823 cells described in (B). (E) Transwell assays were used to measure abilities of migration and invasion of MKN-28 cells as described in Figure 6K. Scale bar, 50 μm. (F) Comparison between protein expression of YTHDF1 in patients with GC and their adjacent normal tissues according to TMA2 dataset (n=28). (G) Representative immunohistochemical images of YTHDF1 expression in primary GC tissues and normal gastric gland from TMA2 (n=28). Scale bar, 50μm. Co-IP, Co-Immunoprecipitation; GC, gastric cancer; TMA, tissue microarray; Data are shown as means ± S.D. ns, no significance.

Data availability statement

The datasets presented in this study can be found in online repositories. The names of

the repository and accession number(s) can be found below: GSE166972.
