# Supplementary figures and images for: The m6A Reader YTHDF1 Facilitates the Tumorigenesis and Metastasis of Gastric Cancer via USP14 Translation in an m6A-Dependent Manner
Source: Front Cell Dev Biol. 2021 Mar 15;9:647702. doi: 10.3389/fcell.2021.647702 (PMC8006284; doi:10.3389/fcell.2021.647702)

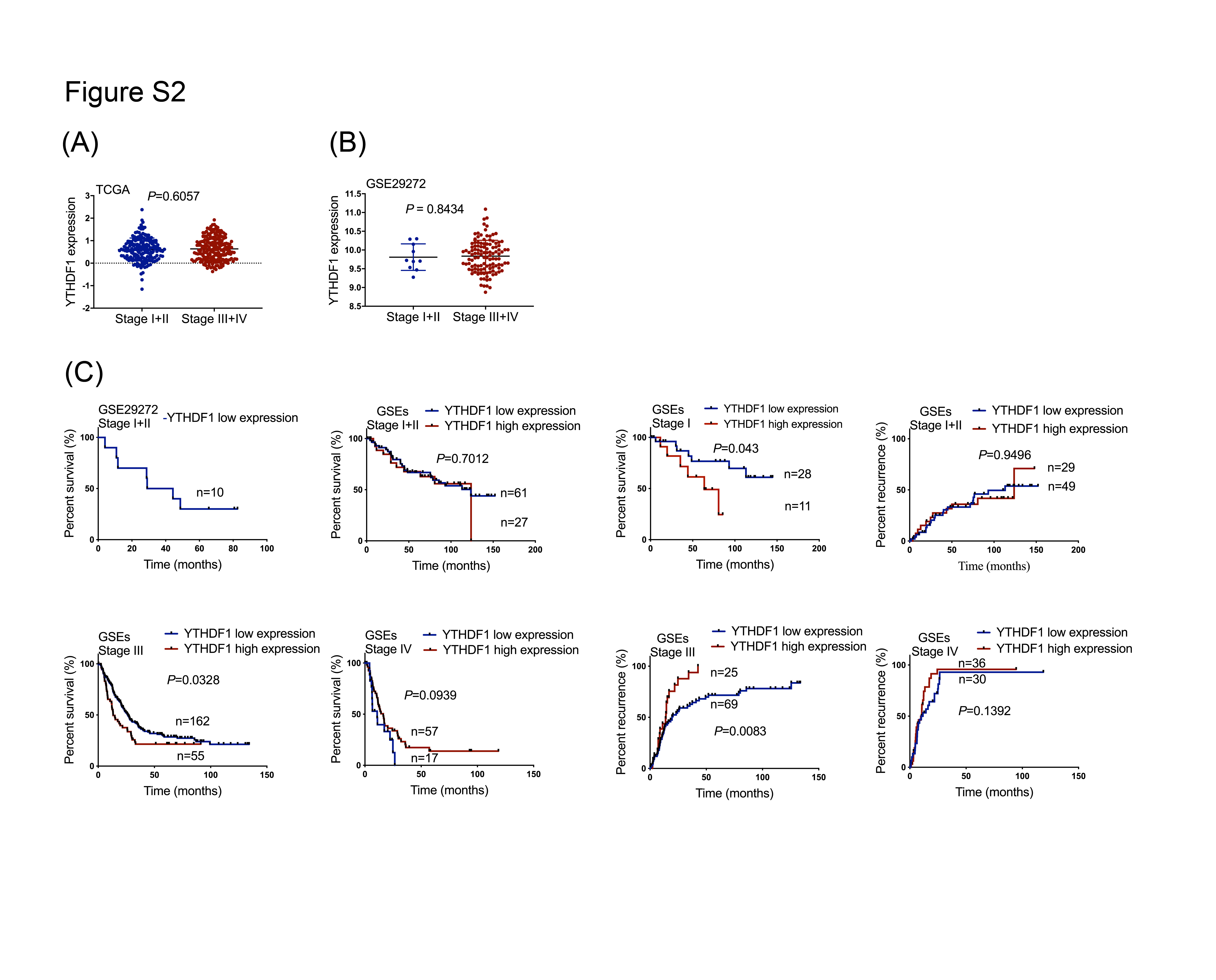

Supplement: Supplementary file 4 [file Image_2.JPEG]

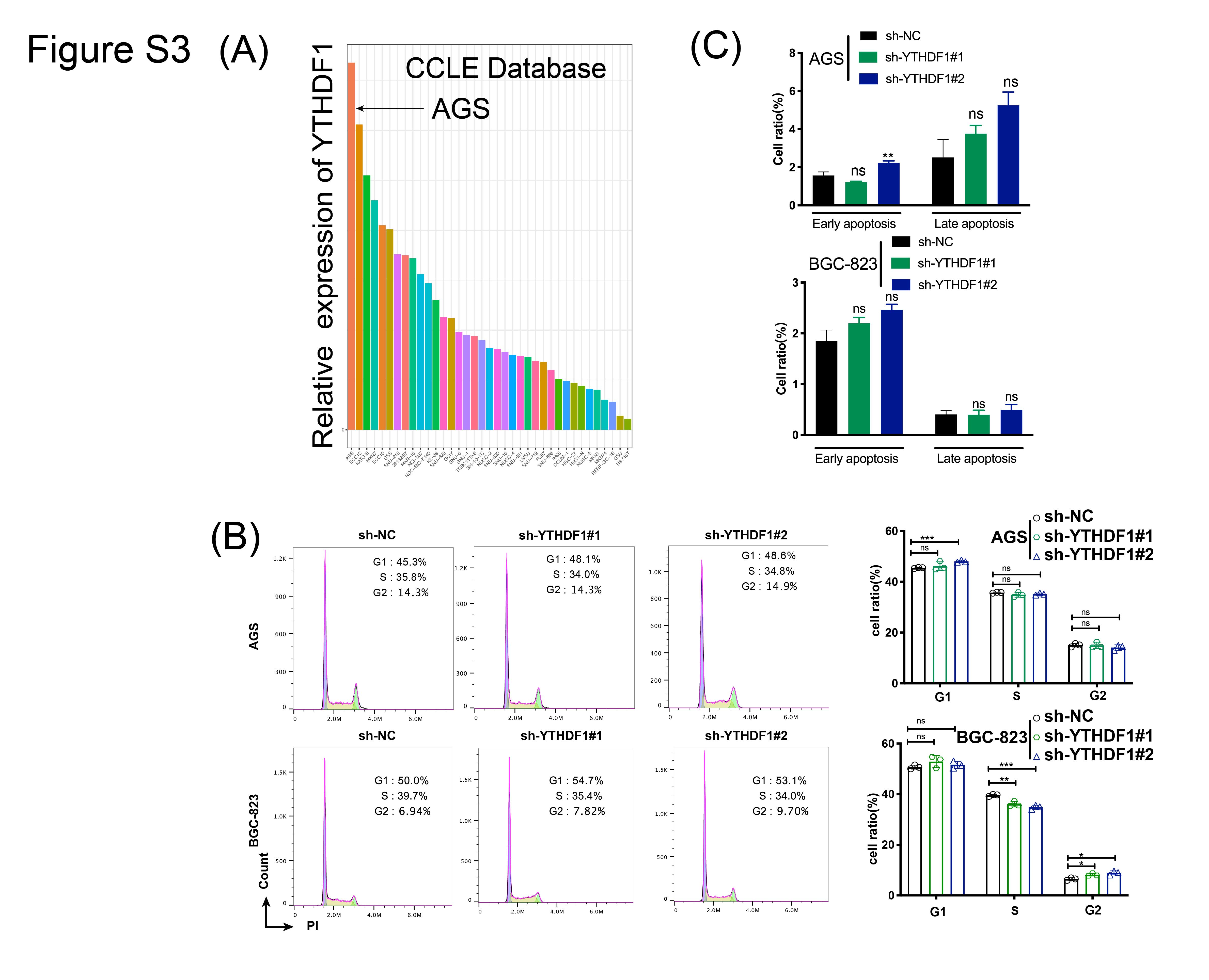

Supplement: Supplementary file 5 [file Image_3.JPEG]

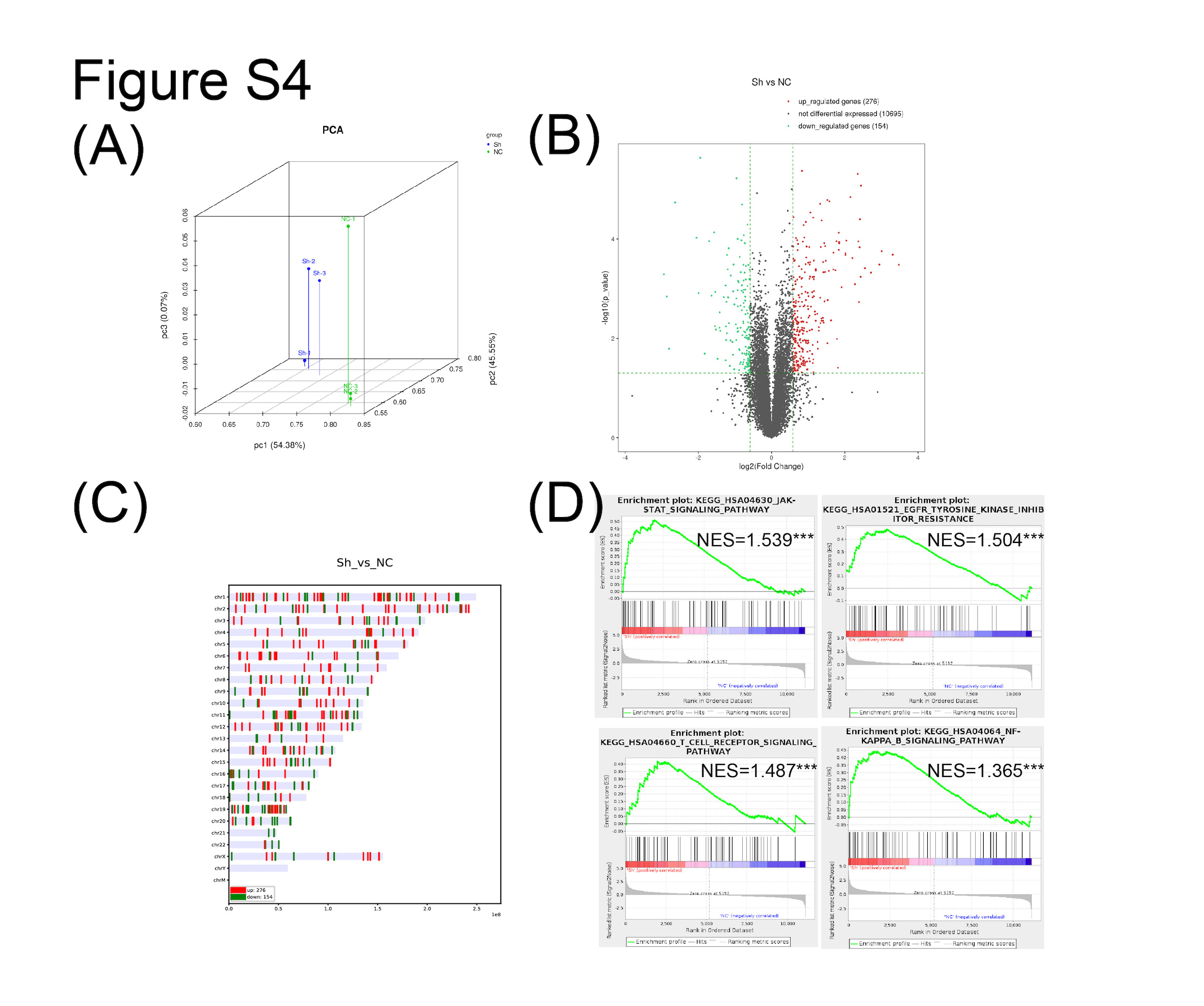

Supplement: Supplementary file 6 [file Image_4.JPEG]

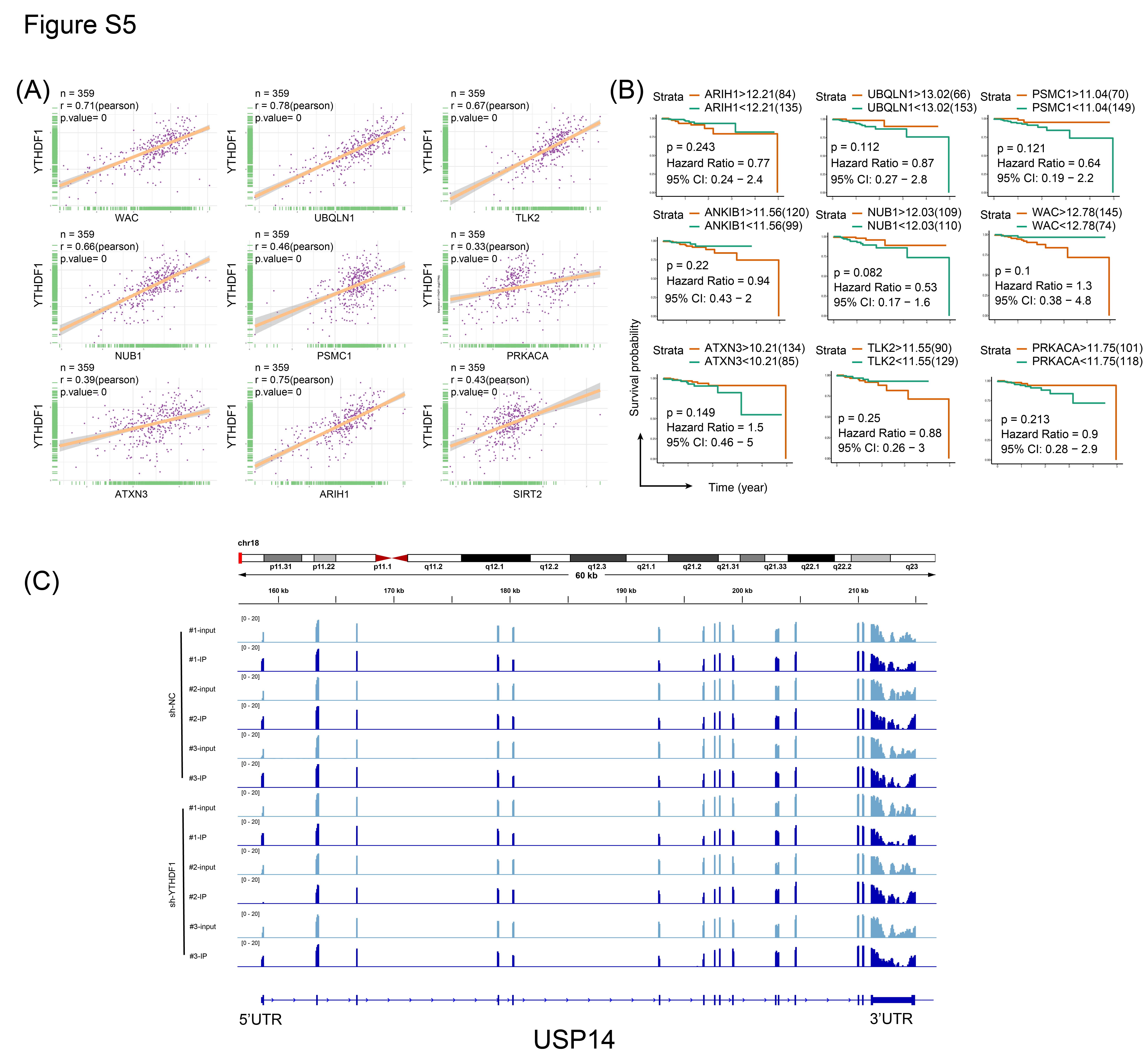

Supplement: Supplementary file 7 [file Image_5.JPEG]

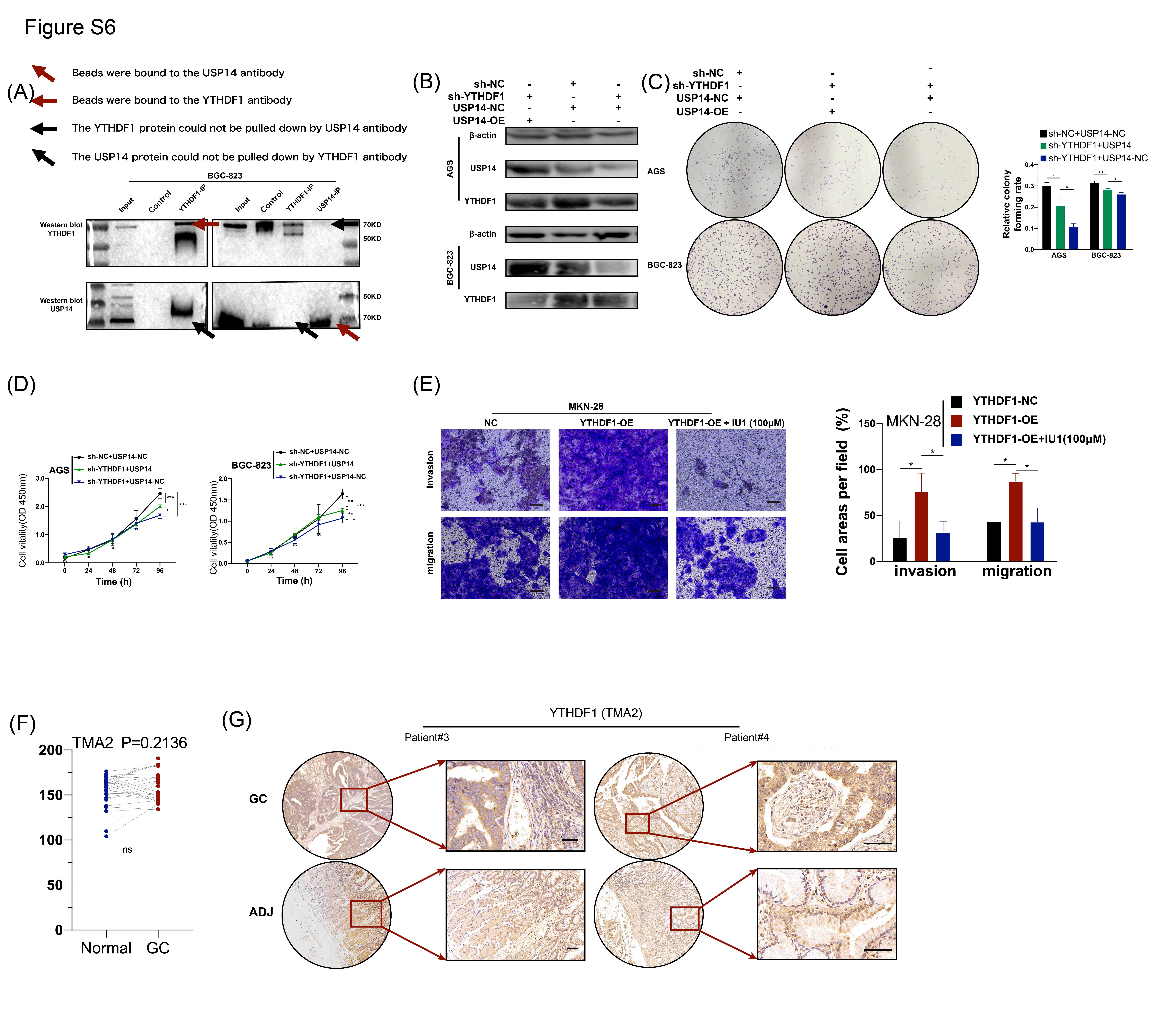

Supplement: Supplementary file 8 [file Image_6.JPEG]
